# A Multitasking Electrical Impedance Tomography System Using Titanium Alloy Electrode

**DOI:** 10.1155/2017/3589324

**Published:** 2017-10-31

**Authors:** Abdalla Salama, Amin Malekmohammadi, Shahram Mohanna, Rajprasad Rajkumar

**Affiliations:** ^1^Department of Electrical and Electronic Engineering, University of Nottingham Malaysia Campus, Semenyih, Selangor, Malaysia; ^2^Faculty of Electrical and Computer Engineering, University of Sistan and Baluchestan, Zahedan, Iran

## Abstract

This paper presents a multitasking electrical impedance tomography (EIT) system designed to improve the flexibility and durability of an existing EIT system. The ability of the present EIT system to detect, locate, and reshape objects was evaluated by four different experiments. The results of the study show that the system can detect and locate an object with a diameter as small as 1.5 mm in a testing tank with a diameter of 134 mm. Moreover, the results demonstrate the ability of the current system to reconstruct an image of several dielectric object shapes. Based on the results of the experiments, the programmable EIT system can adapt the EIT system for different applications without the need to implement a new EIT system, which may help to save time and cost. The setup for all the experiments consisted of a testing tank with an attached 16-electrode array made of titanium alloy grade 2. The titanium alloy electrode was used to enhance EIT system's durability and lifespan.

## 1. Introduction

Electrical impedance tomography (EIT) is a relatively new imaging technique [1, 2]. Developed to overcome some of the disadvantages of existing imaging techniques, it provides flexibility, radiation-free operation, and noninvasive imaging ability and is relatively inexpensive [3–13]. The EIT system operates using low-power signal injection and measurements to reconstruct the estimated cross-section image [5]. It works by detecting and tracking the electrical conductivity changes hidden in a medium such as a pipeline, human body parts, or chemical process [4, 14–19]. The changes in conductivity are measured using several electrodes placed around the body [5, 10, 13, 20–22]. Numerous EIT researchers have studied and attempted to implement a system that can estimate the hidden object structure (shape), which may help to identify the hidden object [4, 13, 23, 24]. Their systems have functioned by controlling the injection and measurement signal parameters such as injection frequency, voltage amplitude, and phase [1, 8, 9, 11, 14, 16, 25–27]. To accomplish the required task of identifying a hidden object, an EIT system that controls and monitors I/O system signals was designed and implemented to improve the image reconstruction capability. However, in studies of EIT applications that have exposed the system to harsh environmental testing conditions, the lifespan of the traditional EIT electrode was reduced as the electrode was damaged [12, 28]. This damage will increase the measurement error [29]. Hence, a durable, flexible, corrosion-resistant electrode is required, particularly for applications that come in direct contact with human skin or tissue or for industrial processes [25, 30]. Therefore, the present EIT system, with 16 titanium alloy electrodes, was developed for use in different applications. Titanium is strong, lightweight, corrosion-resistant, nontoxic (biocompatible), long-lasting, low-cost, and nonferromagnetic [31]. Pure titanium does not have good electrical conductivity, but titanium alloy is a good conductor.

**Figure 1:**
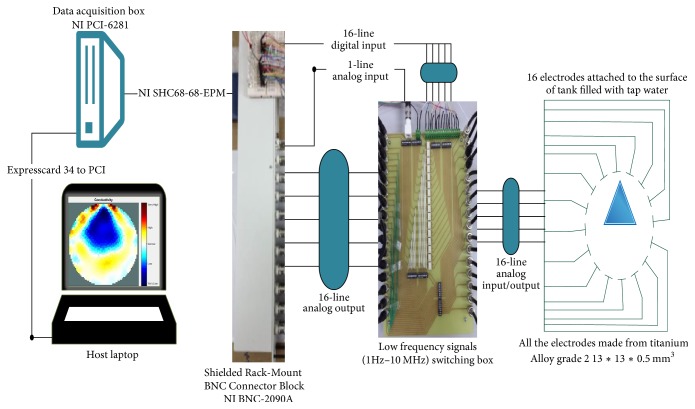
The current structure of the UNMC EIT system.

In this project a new multitasking electrical impedance tomography (EIT) system is designed and implemented to improve the flexibility and durability of an existing EIT system. Here, multitasking means the ability to monitor and control different input/output (I/O) parameters and run LabVIEW and MATLAB software simultaneously building an EIT system to be more flexible and to make it easy to perform multiple computing tasks as presented in Figure 3. The results demonstrate that the new EIT system can be used for different applications without the need to build a new EIT hardware. However, some applications require different electrode sizes, shapes, and materials. Four experiments were conducted. The first experiment examined the capability of the system to detect and track electrically conductive materials with different diameters. The second experiment aimed to detect and monitor air bubbles in water. The third experiment involved the imaging of nonconductive objects and shapes. Finally, the last experiment consisted of detecting chicken fat and skin placed in chicken meat. These experiments show the capability of the system to be used in different applications and fields, such as industrial and medical ones. The results reveal that the developed EIT system can detect an object as small as 1.5 mm in diameter, in a testing vessel 134 mm in diameter. In addition, the results show the capability of the system to estimate various objects and shapes. As shown in Figure 1, the system structure of the EIT system developed at the University of Nottingham Malaysia Campus (UNMC) consists of five main parts: host computer, data acquisition box, BNC connector block, switching box, and a 16-electrode array. Electrical Impedance and Diffuse Optical Tomography Reconstruction Software (EIDORS) was used for all the experiments. EIDORS is an EIT software tool box developed by Andy Adler and is in open-source MATLAB code [32]. Furthermore, a LabVIEW program was implemented to control and monitor the raw I/O signals. The programmable EIT system can be adapted for many of the EIT applications, which may help for different imaging purposes. For example, in some emergency medical situations, a patient must go through several imagining machines for a diagnosis. The time required for these imaging tests will slow down the ability of the doctors to diagnose the diseases; programmable EIT may help to prevent this slowdown in the imaging process.

## 2. Method and Materials

### 2.1. Data Collection

**Figure 2:**
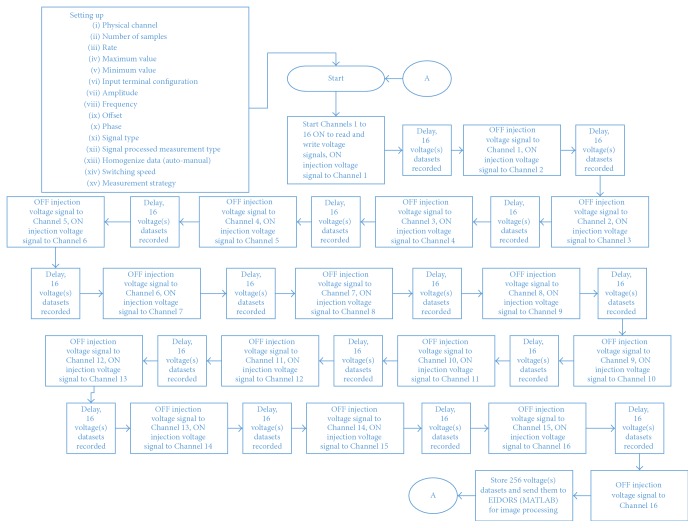
Flowchart of the stages of the process for the programmable EIT system.

The data were recoded for all the experiments using 16 electrodes, with two pipes of the same diameter (14 cm) and different heights. The adjacent (neighbouring) measurement strategy for all the experiments was to match the reconstruction algorithm used by EIDORS, even though this system can support different measurement strategies [19, 32].  Figure 2 illustrates the stages of the process in a flowchart to provide insight into the current EIT system measurement strategy. The system setup was configured based on the applications and the electrode specification. For example, the amplitude was set based on the electrical conductivity environments and the diameter of the region. Therefore, low conductivity requires high signal injection and high conductivity needs a low signal injection. However, the injection amplitude and frequency are limited; the amplitude range is 0.1–10 Vpp, and the frequency range is 1 Hz to 1 MHz. The lowest measurable measurement change, termed code width, was determined by the data acquisition (DAQ) analog-to-digital converter (ADC) resolution and the range of the input signal (maximum value/minimum value). In addition, the input signal resolution for the current DAQ system is 18 bits. Equation (1) shows the formula used to estimate the voltage code width provided by National Instruments.

(1) $$\begin{array}{c} V_{CW}=\frac{\left(\left(\text{Maximum value}\right)-\left(\text{Minimum value}\right)\right)}{2\wedge \left(\text{resolution}\right)} \end{array}$$

(see [35]).

To begin, signals were injected into Channel 1, and the signal data measurements were taken simultaneously from all 16 channels. Then, the signal injection was moved to the next channel (Channel 2), and signal data measurements were recorded from all the channels. This procedure was repeated for each channel, in turn. Furthermore, the number of independent data measurements obtained for a complete dataset *M* is *N* × *N*, where *N* is the total number of channels (16 in this case). Therefore, the total number of independent measurements is 256. Fundamentally, a complete set of data measurements must be performed for every image slide, and the delay time can be controlled; the delay time to be set depends on the measurement sampling rate. Therefore, a high measurement sampling rate requires a longer time to process than a lower sampling rate. In EIT experiments, the measured voltage variances could be calculated, and the variance data were uploaded to the EIDORS for image reconstruction. In fact the reconstructed images are based on the difference in the measurements of peak-to-peak voltages due to adding an object into the background liquid, so the values would be real numbers.

### 2.2. Software and Hardware

**Figure 3:**
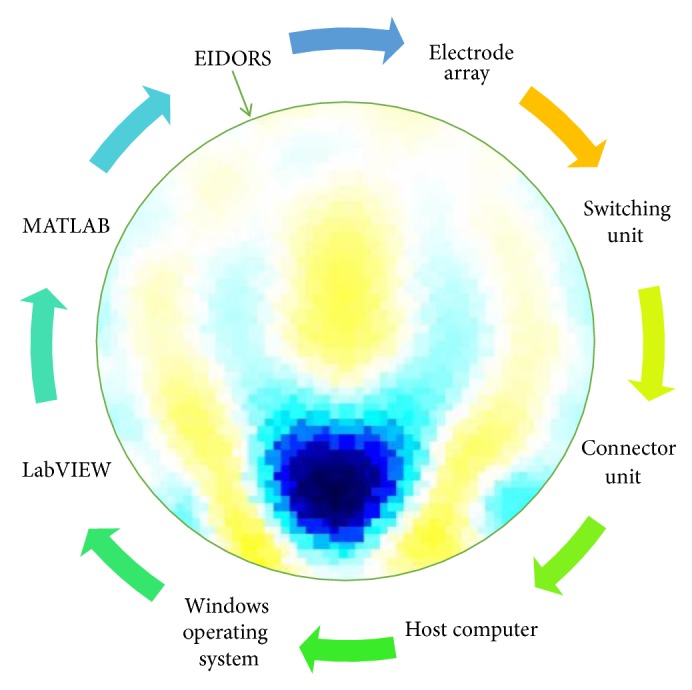
UNMC EIT system operational sequence cycle.

The UNMC EIT system includes hardware and software components. The hardware primarily consists of the host computer, DAQ system, connector unit, switching unit, and electrode array. The software can be divided into three parts: Windows operating system, MATLAB, and LabVIEW. MATLAB was used to reconstruct the image based on the processed data received from the LabVIEW program using EIDORS. The LabVIEW program was designed and implemented to control and monitor the EIT system. Furthermore, the LabVIEW program was developed to control the switching unit to inject and record the signals, process the raw data, and then stream the information to MATLAB for further processing to reconstruct the cross-sectional image. The switching unit connects the DAQ with the electrode array. The switching unit is designed to handle the frequency range of 1 Hz to 1 MHz and that is due to the use of electromechanical low signal relay. Figure 3 shows the continuing sequence stages of cycle flow for the EIT system.

### 2.3. Electrode

**Figure 4:**
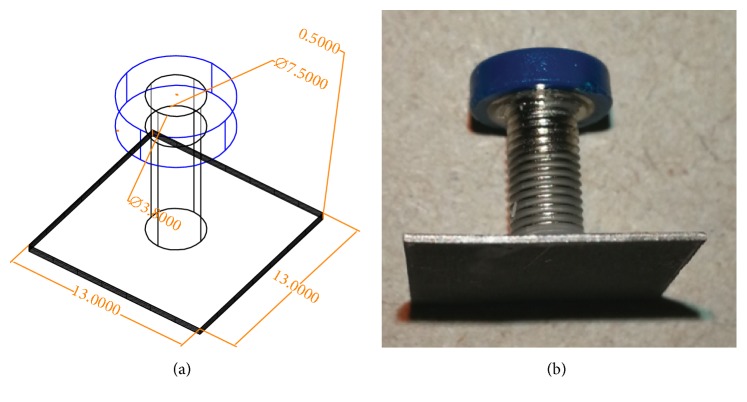
Electrode design layout using AutoCAD (a). The real electrode (b); square contact areas are 13 × 13 × 0.5 mm^3^.

**Figure 5:**
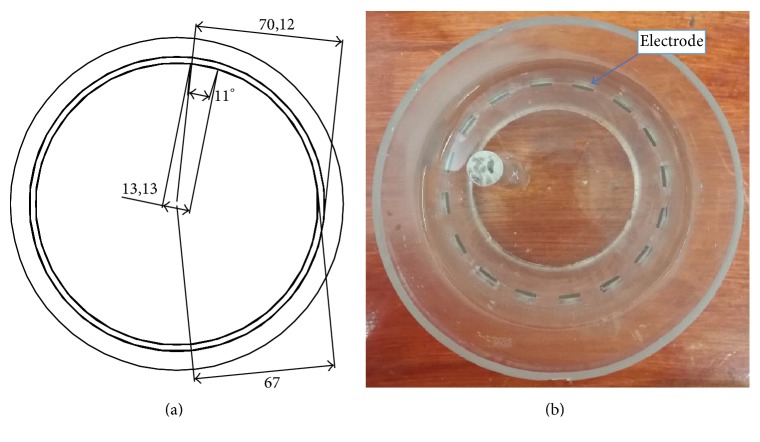
(a) shows the top view dimensions of the testing tank. (b) is the top view of the actual testing tank.

An electrode is an electrical conductor made of metallic material (and could be nonmetallic) used to contact the medium of an object, through which a current, either alternating current (AC) or direct current (DC), enters or leaves a nonmetallic substance medium [10, 19]. Moreover, an electrode array is used in the EIT to detect and track electrical activity. Several material characteristics should be considered to make a suitable electrode for the EIT system; these include conductivity, durability, flexibility, light weight, corrosion resistance, lack of toxicity, long life, nonferromagnetic properties, and low cost. Hence, titanium alloy grade 2 electrodes were designed and implemented as shown in Figure 4. The active area of the titanium alloy grade 2 electrodes is in direct contact with the medium of diameter 13 × 13 × 0.5 mm^3^. The electrode used to apply signals is called the “injection electrode”; the electrode used for measuring signals is called the “measurement electrode.” The same electrode was used for the injection and measurements. In the present EIT system, a plurality of electrodes is arranged in a ring shape with equal spacing as shown in Figure 5. The 16 electrodes were attached approximately 1493.5 mm from the bottom of the testing tank.

## 3. Results and Discussion

The UNMC EIT system was evaluated, including its ability to be used in different applications by tracking and detecting conductive changes of targeted objects. Therefore, several EIT system experiments were conducted, and some of the data results are presented here. Basically, four experiments were conducted to show the aptitude of the EIT system to be adapted for numerous applications. The results of all the experiments (parts) were obtained using an adjacent injection measurement strategy with injection frequency range of 1–1000 kHz. Moreover, the signal measurements used were controlled and monitored using LabVIEW in all the experiments to reconstruct the cross-sectional image. The variance data were calculated from the recoded measurement signals for all the experiments. For the first three experiments (parts), an electrode with contact active area dimension of 13 × 13 × 0.5 mm^3^ was employed; for the fourth experiment, the electrode active area dimension was 14 × 14 × 0.5 mm^3^. All the experiments were conducted using two testing tanks (cylindrical shape), both of 140 mm diameter; one tank was 280 mm high and the other was 50 mm high. The 280 mm high tank was used for the first three experiments, and the 50 mm high tank was used for the fourth experiment. A titanium alloy electrode with an active contact area of 13 × 13 × 0.5 mm^3^ was employed for the 280 mm high vessel, and a 14 × 14 × 0.5 mm^3^ electrode was used for the 50 mm high tank. The first experiment showed the ability of the system to detect and track electrically conductive and nonconductive objects of different diameters when they were placed in a range of positions in the testing vessel filled with 3200 ml of tap water, one after another. For the third experiment, which was conducted to detect and track an air bubble, the air hoses were located in various positions in the testing vessel. The conductivity of the tap water, which was measured using an electrical conductivity meter, was approximately 97 *μ*S/cm at 27°C. The last experiment was performed to detect chicken fat and skin in a tank filled with chicken meat chunks. For all the experiments, the first measurements were obtained when only tap water or chicken meat was in the tank. For the second measurements, the electrically conductive and nonconductive objects were placed in different locations inside the testing vessels. Then, the two measurements were compared and processed and saved using LabVIEW; then, they were streamed to MATLAB (EIDORS) for further processing for image reconstruction. Furthermore, a voltage input signal range of ±2 V was used for all the experiments. The results obtained with the UNMC EIT system are presented in Tables 1–6 and Figure 7. The conductive objects were a stainless-steel rod and steel wire rope with diameters 26, 12, 4, 3, 2, and 1.5 mm. The nonconductive objects were a plastic rod, Styrofoam, and plastic wire rope with diameters 25, 12, 7, 4, and 2 mm. Some of the targeted objects, whether conductive or nonconductive, were placed in a few different locations inside the testing vessel and exposed to various injection signal frequencies: 300, 400, 500, 900, and 1000 kHz. Every table includes the actual object location and the reconstructed image of the object. The five primary targeted object placement locations for all the experiments are listed below:

1. Near electrodes 15, 16, 1, and 2
2. Near electrodes 3, 4, 5, and 6
3. Near electrodes 6, 7, 8, and 9
4. Near electrodes 11, 12, 13, and 14
5. In the centre.

**Figure 6:**
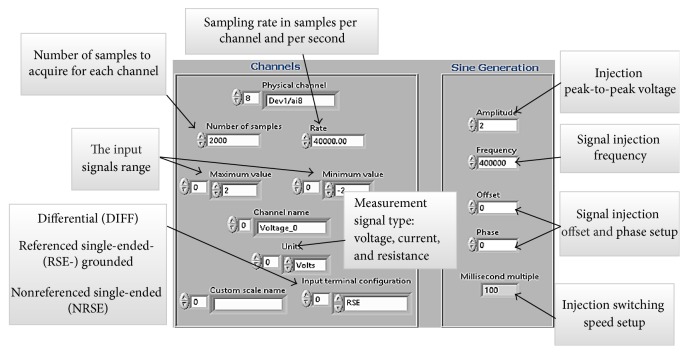
Channel configuration and signal generator configuration controls.

Figure 6 shows the typical analog I/O signal configuration setup window for all the experiments. However, these settings can be modified based on the application requirements.

Table 1 shows the 2D reconstruction images of three electrically conductive objects with three different diameters placed in several locations. The input signal range was max 3–min −3, and the injected frequency, as shown in this table, was 400 kHz and 500 kHz. The colour scale illustrates the states of the electrical conductivity changes. The red colour means that the electrical conductivity increased in that area, and the blue colour signifies that the electrical conductivity in that area dropped. As observed, the steel rod diameter of 26 mm was placed in the four stated locations and tracked and imaged. Similarly, the electrically conductive object with a diameter of 12 mm was placed in the locations shown and tracked and imaged. However, image background noise was present and at an inconsistent level; this noise did not affect the reconstructed image of the object, as shown in the table. In contrast, the steel wire rope with a diameter of 4 mm was placed in four locations and detected and imaged; the reconstructed image when the object was located in the centre of the testing vessel had a high amount of distortion, as shown. Therefore, the injected excitation frequency was increased from 400 kHz to 500 kHz, which improved the reconstruction image of the object when it was in the centre.

Table 2 also shows the 2D reconstruction images of three electrically conductive objects of three different diameters placed in several locations, but with the objects smaller in diameter than the objects in Table 1. As observed, the steel wire rope with a diameter of 4 mm was placed in four stated locations and tracked and imaged using 400 kHz as the excitation frequency. However, when the object was positioned in the central location, the reconstructed image of the object shifted. This object-shifting problem was solved by increasing the injected excitation frequency from 400 kHz to 500 kHz; the reconstructed image is displayed in the table. Moreover, the steel wire rope with a diameter of 3 mm, which was placed in the four stated locations, was tracked and imaged using 500 kHz as the excitation frequency. In addition, when the 2D reconstructed image object was placed in the centre, a high distortion occurred, even when using 500 kHz for the injection excitation frequency. However, the image distortion was solved by decreasing the input signal range from max 3–min −3 to max 1–min −1, which increased the measurement sensitivity of the signal. The last column of the table shows the results of the smallest electrically conductive object in this experiment, with a diameter of 1.5 mm. As shown, the current EIT system can image and track the object, even though background noise is present and not consistent, but it does not affect the detection of the aimed object, as shown. Therefore, this EIT system potentially can detect electrical conductivity changes from objects as small as 1.5 mm in a testing tank with a diameter of 140 mm as shown in the last column of Table 2.

Table 3 shows the 2D reconstruction images of three electrically nonconductive objects with three different diameters placed in several locations. Plastic rods with diameters of 25 mm, 14 mm, and 7 mm were placed in four locations and tracked and imaged using 0.4 MHz as the stimulation frequency, with an input signal range of max 3–min −3. Although the volume of the reconstructed image background noise is observed and not invariable, it does not affect the reconstructed image of the aimed object, as demonstrated.

Table 4 displays the 2D reconstruction images of two electrically conductive plastic ropes with two different diameters placed in four different regions. The plastic rope with diameters of 4 mm and 2 mm was identified and imaged using 0.4 MHz as the excitation frequency with a range of input signals of max 3–min −3. The effects of the changes in object diameter on the reconstructed images were noticed and changed slightly depending on the object width. In contrast, the artefact of the reconstructed images was noticeable and did not affect the reconstructed images of the targeted objects, as demonstrated.

The results in Tables 1–4 show the capability of the first UNMC EIT system to track and image electrically conductive and nonconductive objects with stated diameters which were placed in a testing tank filled with tap water. As seen, the electrically conductive materials required more calibration and configuration setup than the nonconductive objects, particularly when the targeted object was smaller. It also was observed in some cases that the reconstructed images of the electrically conductive object were good in most targeted object locations and became weak in the centre of the testing tank. This may be because the electrical conductivity at high frequency becomes low; hence, the current easily passes through the targeted object when the targeted object diameter becomes smaller and farther from the electrodes. However, this issue was solved in two ways. The first solution was to increase the injected excitation frequency from 0.4 MHz to 0.5 MHz. The second was to decrease the input signal range from max 3–min −3 to max 1–min −1 to increase the measurement signal sensitivity.

Table 5 shows the results of the second experiment. The first measurements were obtained when only tap water was used, and the air hose was placed in a fixed location. The second measurements were taken when the air was pumped through the air hose, which was fixed in different locations inside the testing tank. Then, the first and second measurements were compared to reconstruct 2D images showing the air bubble flow location. As it is known that air bubbles' flow is not steady, the three reconstructed images were unsteady for the samples presented for each location as displayed. As observed, the air bubble flow area was located and imaged using 0.5 MHz as the excitation frequency, with input signals ranging within max 3–min −3. However, as seen when the air was pumped into the central location, the reconstructed image of the air bubble area changed and varied (being not stable). This may be because of the air bubble flow behaviour, which appeared to migrate into the testing tank wall regions.

Table 6 shows the results of the third experiment, and it illustrates the ability of the system to detect and reconstruct the images of non-electrically conductive object shapes that were placed in the testing vessel filled with 2250 ml of tap water. The first column shows the Styrofoam real shapes, and the centre column demonstrates the reconstructed cross-section images for each aimed target shape. The last column shows the analog I/O signal main configuration setup parameters for each targeted object shape. The first object, which was made of Styrofoam, was carved into a circle shape, and it was placed near electrodes 15, 16, 1, and 2. The reconstructed image shows the ability of the system to estimate the targeted object shape; however, it is not very accurate because the background noise was high, particularly on the left side of the imaged area, as seen in the dark red spot. The second object was carved into a square shape, and it was placed near electrodes 14, 15, 16, 1, 2, and 3. The target was detected and imaged as a square shape with low defects, even though background noise was present. The third object was carved as a square shape as well, but with a smaller diameter size than the previous object, and was placed near electrodes 15, 16, 1, and 2. In addition, the small square object was detected and imaged, but with a low defect. The fourth object was carved as a triangle shape and placed near electrodes 16 and 1. As seen in the table, the triangle shape was reconstructed with a low amount of background noise. Furthermore, a smaller triangle object was carved and imaged as shown; it was placed near electrodes 16 and 1. The L-shaped object, which was placed near electrodes 15, 16, 1, 2, 3, and 4, was imaged. As observed, the structural details of the reconstructed object are poor, particularly the object side towards the centre of the testing tank. The C-shaped object, which was located near electrodes 14, 15, 16, 1, 2, and 3, was identified with few defects. Finally, the E-shaped object, which has a complex shape structure, was compared with the previous object shapes and was placed near electrodes 14, 15, 16, 1, 2, and 3; it was recognised but had a high number of defects.

**Figure 7:**
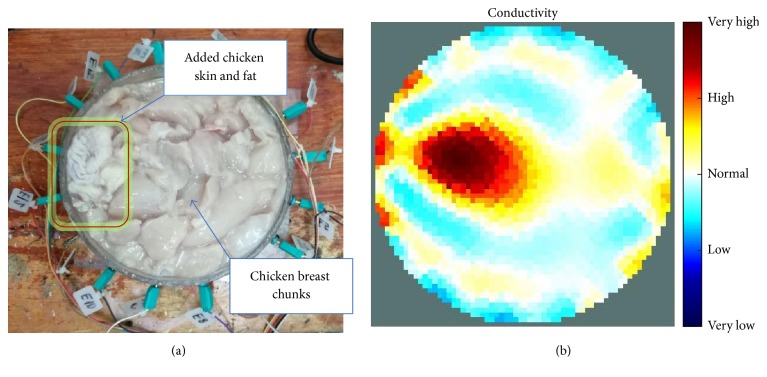
(a) Chicken skin and fat location in the vessel filled with raw chicken breast chunks. (b) 2D reconstructed image of the chicken skin and fat.

Figure 7 shows the results of the fourth experiment, which employed a testing tank with a height of 50 mm and electrodes and contact active area dimension of 14 × 14 × 0.5 mm^3^. The first measurements were taken using a testing tank filled with chicken breast chunks and with an electrical conductivity level of 1184.04 *μ*S/cm. The second measurements were taken from the tank when chicken fat and skin (soaked in brackish water) with an electrical conductivity of 2870.4 *μ*S/cm were located near electrodes 11, 12, 13, and 14. Then, the measurement data changes were processed to identify the location of the chicken skin and fat. As seen, the UNMC EIT prototype system was able to find and image the electrical conductivity changes in the tank.

The experimental results show four different use cases of our UNMC EIT system. Moreover, the results demonstrate in Tables 1–6 and Figure 7 that the UNMC EIT prototype system has the flexibility and capability to be used in different EIT applications. The system potentially can detect electrical conductivity changes from objects as small as 1.5 mm in a testing tank with a diameter of 140 mm.  Table 7 shows the developed EIT system in comparison to some of the latest EIT systems.

## 4. Conclusion

A multitasking EIT system implemented using 16 titanium alloy electrodes overcomes some of the drawbacks of the conventional EIT system, including flexibility and durability. The experiments were conducted using two different testing tanks with two different electrode array dimensions. Furthermore, the results show that the system can detect an object with a diameter as small as 1.5 mm that is placed in the testing tank with a diameter of 134 mm. The results show that the system can be adapted for different EIT applications; the results are validated by conducting four experiments. Moreover, the flexibility of the UNMC EIT system can be adapted for different applications by controlling and monitoring the configuration of I/O signals such as frequency, voltage amplitude, signal type, offset, sample rate, and code width. The system shows good accuracy to locate and image cross-section objects with simple shapes such as circle, square, and triangle. However, most of the reconstructed images have background noise, although this does not affect the object detection and tracking.

## Figures and Tables

**Table 1:** Conductive objects at different positions; reconstructed images for three different conductive object diameters (26 mm, 12 mm, and 4 mm).

Targeted object, real location, and injected frequency with input signal range (max 3–min –3)	The object is a cylindrical stainless-steel rod (26 mm diameter)	The object is a cylindrical stainless-steel rod (12 mm diameter)	The object is steel wire rope (4 mm diameter)
15, 16, 1, 2 (0.4 MHz) 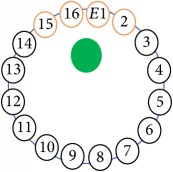	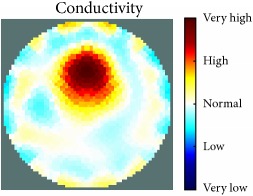	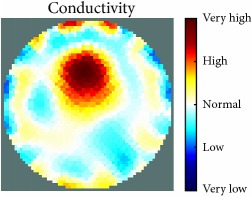	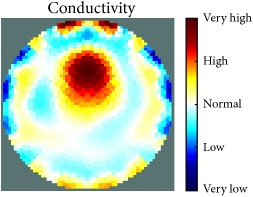
7, 8, 9, 10 (0.4 MHz) 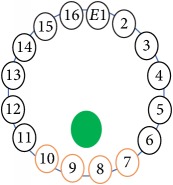	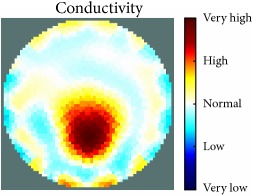	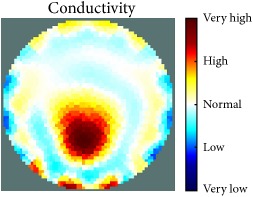	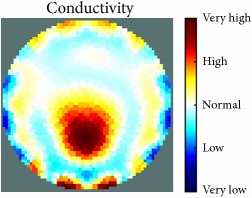
11, 12, 13, 14, (0.4 MHz) 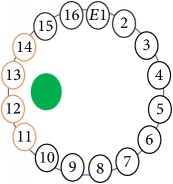	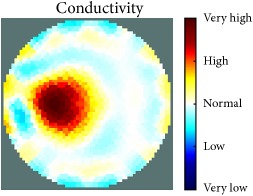	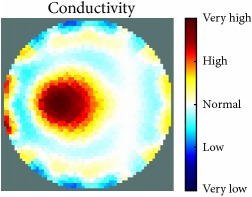	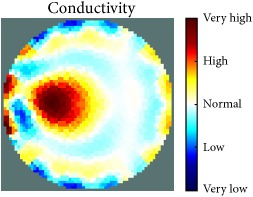
Centre (0.4 MHz) 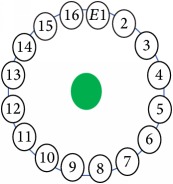	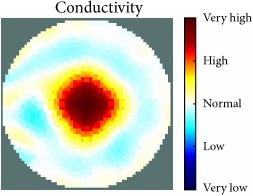	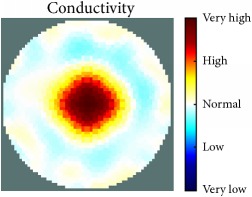	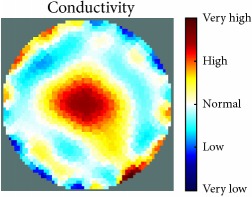
Centre (0.5 MHz) 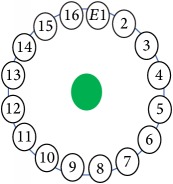			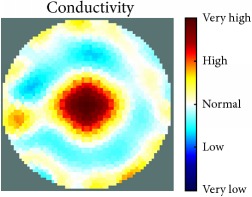

**Table 2:** Conductive objects at different positions; reconstructed images for three different conductive object diameters (3 mm, 2 mm, and 1.5 mm).

Targeted object real location	The object is steel wire rope (3 mm diameter)	The object is steel wire rope (2 mm diameter)	The object is steel wire rope (1.5 mm diameter)
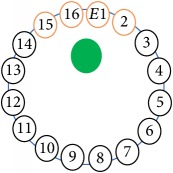	0.4 MHz (max 3–min −3) 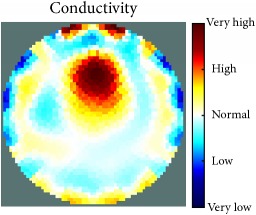	0.5 MHz (max 3–min −3) 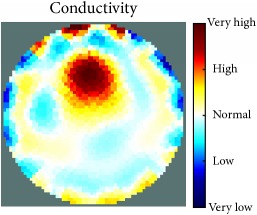	0.5 MHz (max 1–min −1) 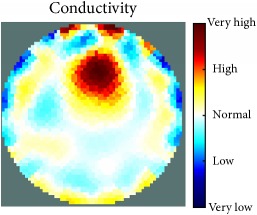
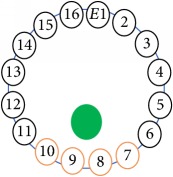	0.4 MHz (max 3–min −3) 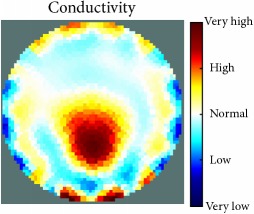	0.5 MHz (max 3–min −3) 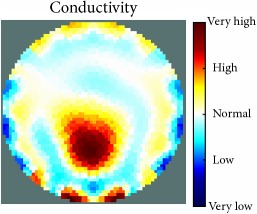	0.5 MHz (max 1–min −1) 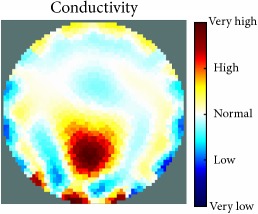
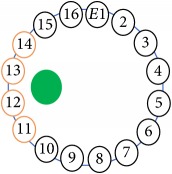	0.4 MHz (max 3–min −3) 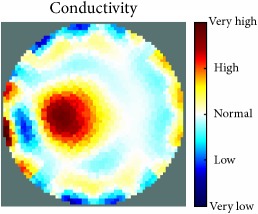	0.5 MHz (max 3–min −3) 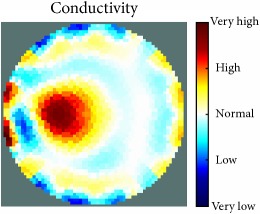	0.5 MHz (max 1–min −1) 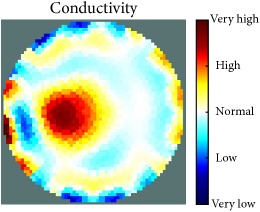
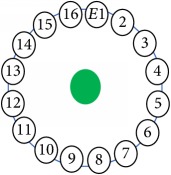	0.4 MHz (max 3–min −3) 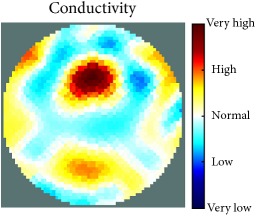	0.5 MHz (max 3–min −3) 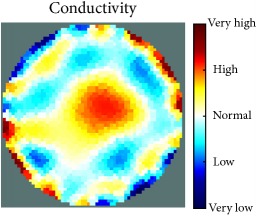	0.5 MHz centre (max 1–min −1) 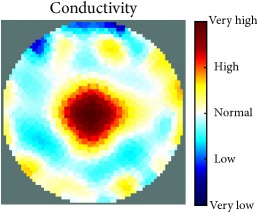
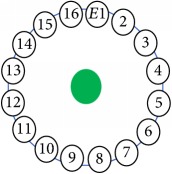	0.5 MHz centre (max 3–min −3) 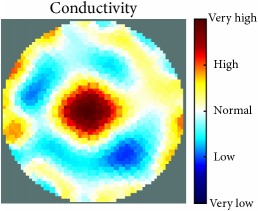	0.5 MHz centre (max 1–min −1) 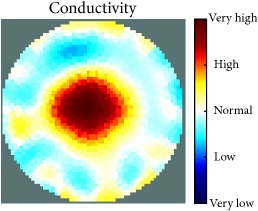	

**Table 3:** Nonconductive objects at different positions; reconstructed images for three different nonconductive object diameters (25 mm, 14 mm, and 7 mm).

Targeted object, real location, and injected frequency with input signal range max 3–min −3	The object is a cylindrical plastic rod (25 mm diameter)	The object is a cylindrical plastic rod (14 mm diameter)	The object is a cylindrical rod (7 mm diameter)
15, 16, 1, 2 (0.4 MHz) 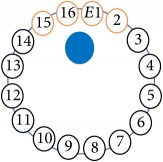	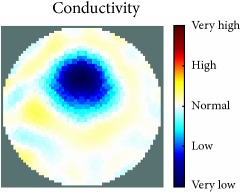	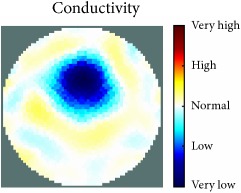	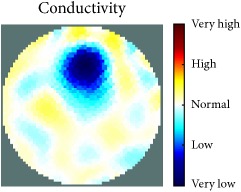
7, 8, 9, 10 (0.4 MHz) 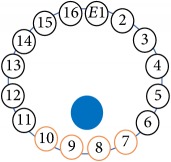	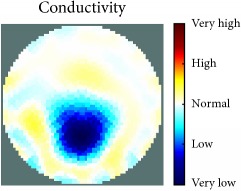	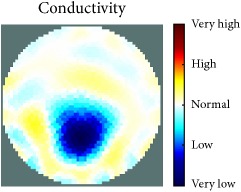	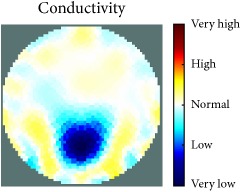
11, 12, 13, 14 (0.4 MHz) 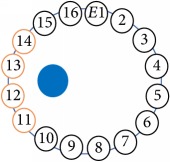	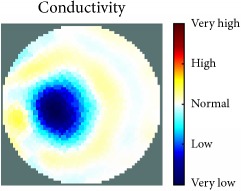	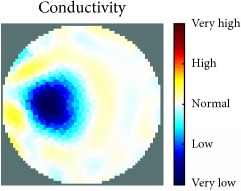	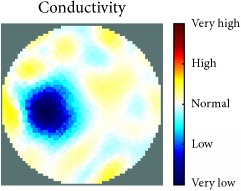
Centre (0.4 MHz) 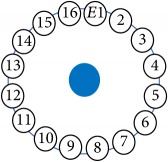	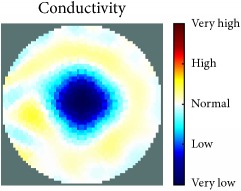	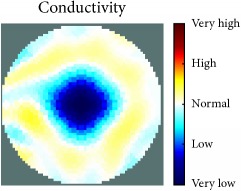	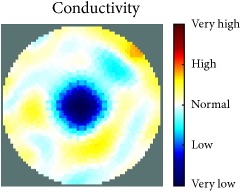

**Table 4:** Nonconductive objects at different positions; images for two different nonconductive object diameters (4 mm, 2 mm).

Targeted object, real location, and the injected frequency with input signal range max 3–min −3	The object is plastic rope (4 mm diameter)	The object is plastic rope (2 mm diameter)
15, 16, 1, 2 (0.4 MHz) 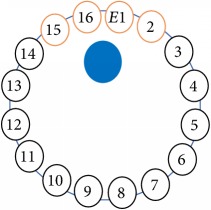	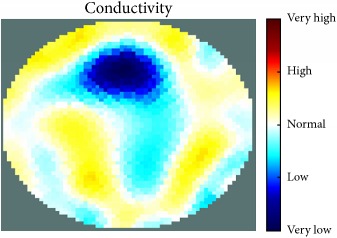	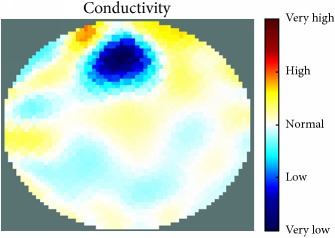
7, 8, 9, 10 (0.4 MHz) 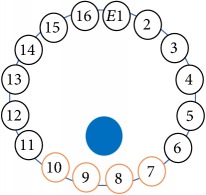	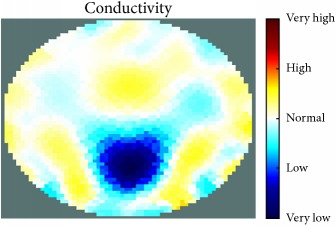	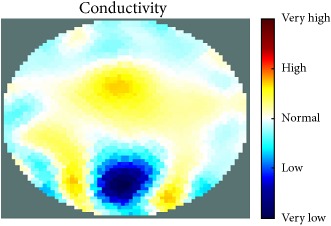
11, 12, 13, 14 (0.4 MHz) 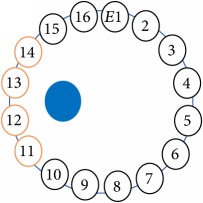	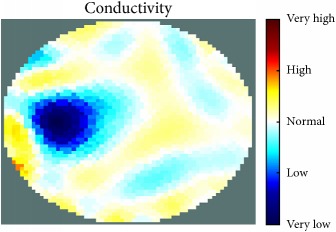	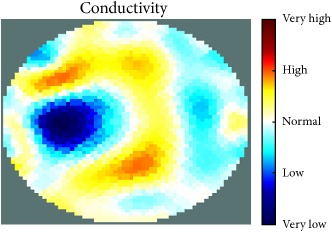
Centre (0.4 MHz) 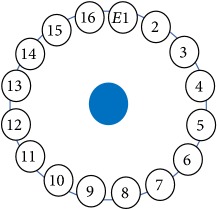	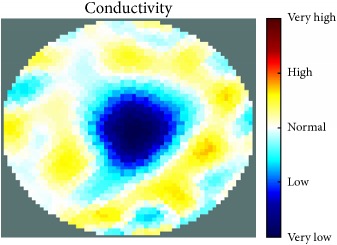	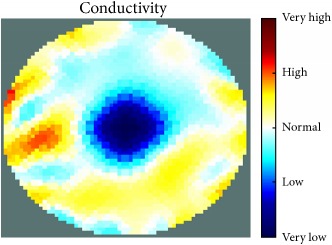

**Table 5:** Air hose placed at different positions; reconstructed cross-section images for five different air hose setups.

Air pumped area targeted real location and the injected frequency with input signal range max 3–min −3	Sample 1	Sample 2	Sample 3
15, 16, 12 (0.5 MHz) 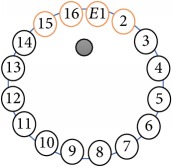	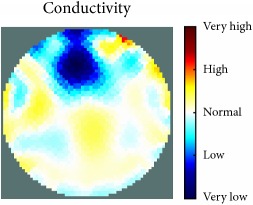	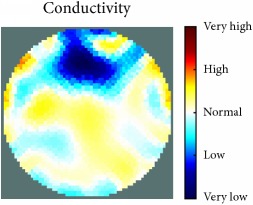	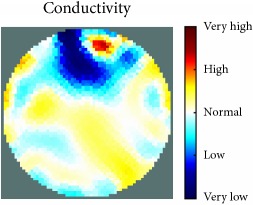
11, 12, 13, 14 (0.5 MHz) 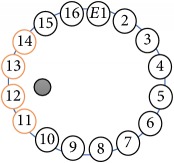	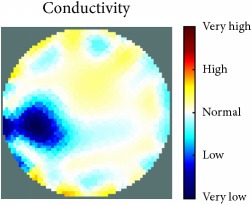	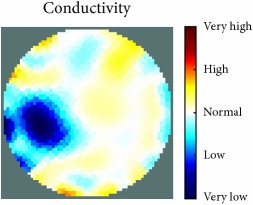	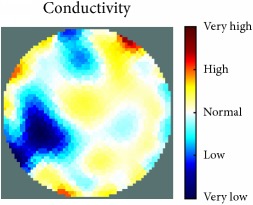
7, 8, 9, 10 (0.5 MHz) 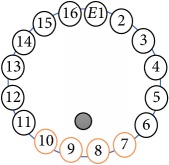	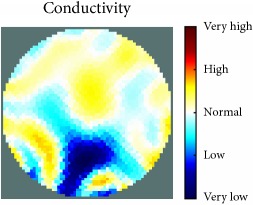	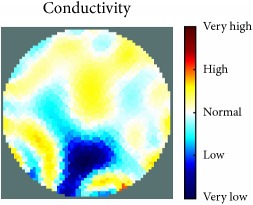	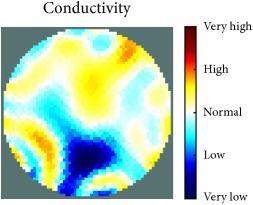
3, 4, 5, 6 (0.5 MHz) 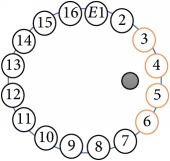	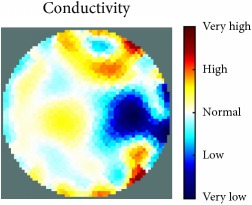	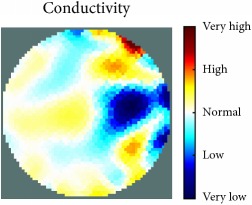	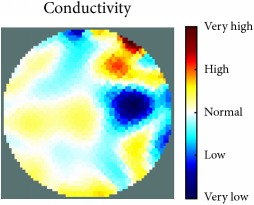
Centre (0.5 MHz) 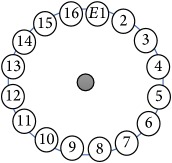	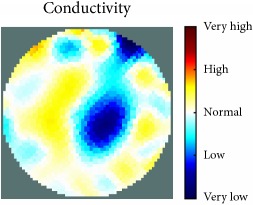	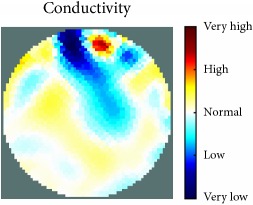	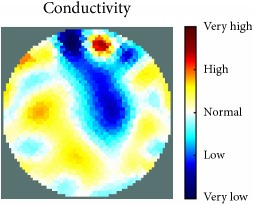

**Table 6:** Nonconductive objects (Styrofoam) with different shapes; reconstructed images for different nonconductive object shapes.

Targeted object shape, made of Styrofoam	The 2D reconstructed image	The analog I/O signal main configuration setup
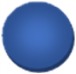	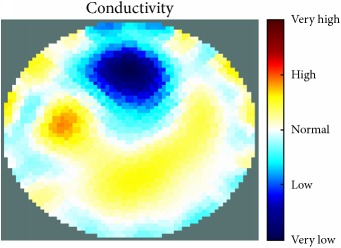	Amplitude: 2 V Frequency: 300 kHz Offset: 0 Number of samples: 2000 Rate: 40000 Maximum value: 3 Minimum value: −3

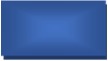	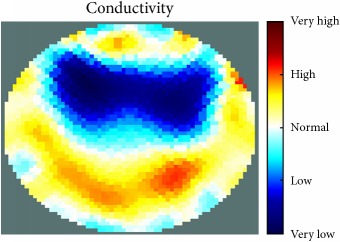	Amplitude: 2 V Frequency: 1 MHz Offset: 0 Number of samples: 2000 Rate: 40000 Maximum value: 1 Minimum value: −1

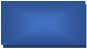	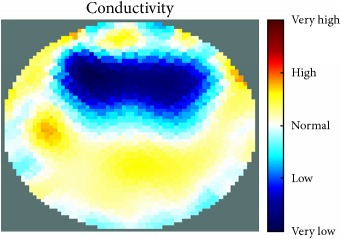	Amplitude: 2 V Frequency: 1 MHz Offset: 0 Number of samples: 2000 Rate: 40000 Maximum value: 1 Minimum value: −1

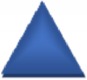	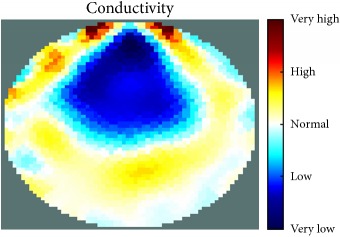	Amplitude: 2 V Frequency: 1 MHz Offset: 0 Number of samples: 2000 Rate: 40000 Maximum value: 0.5 Minimum value: −0.5

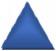	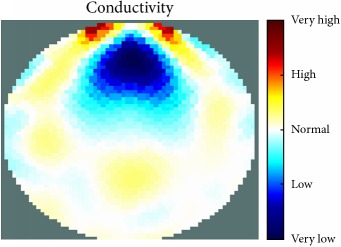	Amplitude: 2 V Frequency: 1 MHz Offset: 0 Number of samples: 30000 Rate: 40000 Maximum value: 0.5 Minimum value: −0.5

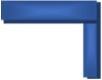	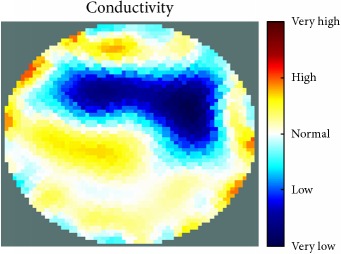	Amplitude: 2 V Frequency: 900 kHz Offset: 0 Number of samples: 2000 Rate: 40000 Maximum value: 1 Minimum value: −1

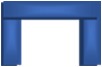	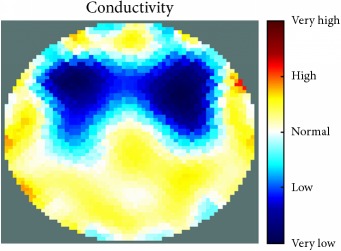	Amplitude: 2 V Frequency: 1 MHz Offset: 0 Number of samples: 2000 Rate: 40000 Maximum value: 0.2 Minimum value: −0.1

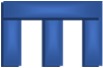	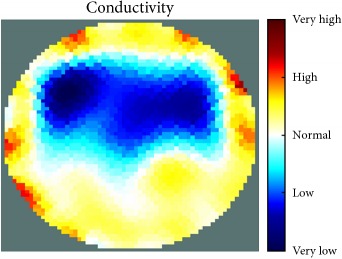	Amplitude: 2 V Frequency: 1 MHz Offset: 0 Number of samples: 2500 Rate: 40000 Maximum value: 0.2 Minimum value: −0.1

**Table 7:** Recent EIT systems and comparison results.

EIT System Developer	Nottingham University	Dartmouth College [33]	KAIST University [16]	Aachen University [27]	Maltron Sheffield MK 3.5	Mobecomm MEIK	Carnegie Mellon University [34]
System	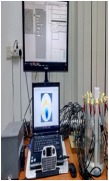	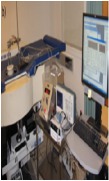	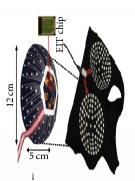	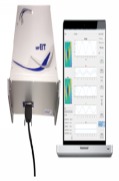	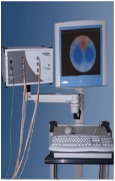	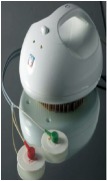	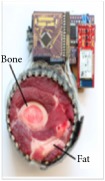

Dimension	140 mm diameter	600 mm	300 × 250 × 50 mm^3^	N/A	N/A	160 × 180 × 100 mm^3^	200 mm diameter

Image dimension	2D	3D	3D	2D	2D	2D	2D

Electrodes	16 (1 layer)	64 (4 layers)	92 (flexible)	16 (2 layers)	8 (1 layer)	256 (planar)	8, 16, 32 (1 layer)

Electrode materials	Copper, titanium alloy	N/A	N/A	N/A	N/A	N/A	Stainless steel

Frequency	1 Hz–1 MHz	10 kHz–10 MHz	100 Hz–100 kHz	60 kHz–1 MHz	2 kHz–1620 kHz	10 kHz, 50 kHz	40 kHz

Multifrequency	Yes	Yes	Yes	Yes	Yes	No	No

Amplitude	0.1–10 V, 5 mA	N/A	10 *μ*A–400 *μ*A	N/A	N/A	0.5 mA	0–6 V, 300 *μ*A

Multiamplitude	Yes	No	No	No	N/A	No	Yes

SNR	SINAD 8–70 dB depends on channels and the injection frequency	94 dB	90 dB	109.6 dB	N/A	N/A	N/A

Minimum detectable size	1.5 mm (in 140 mm)	N/A	5 mm (in 120 mm)	N/A	N/A	3–5 mm	38.1 mm (in 200 mm)

Measurement type	Single-ended	Differential	Differential	Differential	Differential	N/A	Differential

Processed signals	RMS, variance, standard deviations	N/A	N/A	N/A	N/A	N/A	RMS

Imaging device	Computer	Computer	Mobile device	Computer	Computer	Computer	Computer

Year	2016	2015	2015	2016	2014–2016	2010	2016
